# Open Partial Cystectomy of Intramural Bladder Leiomyoma With Unfavorable Position: A Rare Case Report

**DOI:** 10.7759/cureus.12965

**Published:** 2021-01-28

**Authors:** Aiman Al Solumany, Yasser Alobairi, Mohammed Abuzenada, Omar A Sulaiman, Mutaz Fatani

**Affiliations:** 1 Urology, King Fahad General Hospital, Jeddah, SAU; 2 Urology, Umm Al-Qura University, Makkah, SAU

**Keywords:** leiomyoma, bladder tumor, cystectomy

## Abstract

Benign tumor of the bladder (Leiomyoma) is a rare condition representing less than 0.5% of all bladder tumors. A 50-year-old female presented with asymptomatic hematuria for three months. Abdomen and pelvic magnetic resonance imaging (MRI) revealed a large intraluminal mass originating from the submucosal layer of the left anterolateral wall of the urinary bladder. An ultrasound-guided biopsy was performed, and a histopathological examination showed a spindle cell tumor. Cystoscopy revealed a massive indentation in the left posterolateral site of the urinary bladder extending to the bladder's dome. The patient underwent open partial cystectomy. At the follow-up, the patient was evaluated, and there was no hematuria or other complaints. Open partial cystectomy is the treatment of choice in an intramural tumor with an unfavorable position and difficulty in recognition.

## Introduction

Bladder leiomyomas are rare benign mesenchymal neoplasms and represent less than 0.5% of all bladder tumors [1]. Bladder leiomyoma incidence is three times more common in females than in males. Usually, it occurs in the fourth and fifth decades of life [2]. Patients with bladder leiomyomas may be asymptomatic, but most of them present with obstructive and irritative symptoms (49% and 38%, respectively) and hematuria (11%). The etiology of these benign tumors is still unknown. Several theories still discuss the origins of bladder leiomyomas, such as embryonic rest tumors, hormonal disturbances, localized infection, fibroid resembling uterine leiomyoma, and post-inflammatory myomatous metaplasia [3]. Several ways of diagnosing these tumors are available and include ultrasound, computerized tomography (CT), magnetic resonance imaging (MRI), and cystoscopy, but histopathology is the technique used for a definitive diagnosis [4]. Clinical treatments differ according to the size and anatomical location of the tumors. Suppose the tumor is a small and endovesical leiomyoma, in that case, it can be treated with transurethral resection of the bladder tumor (TURBT). Still, in cases of large and intramural or extravesical tumors, it is more effective to be treated with segmental resection and partial cystectomy [5]. In general, the condition has a good prognosis [1]. In this report, we present a case of a middle-aged woman with asymptomatic hematuria.

## Case presentation

**Figure 1:**
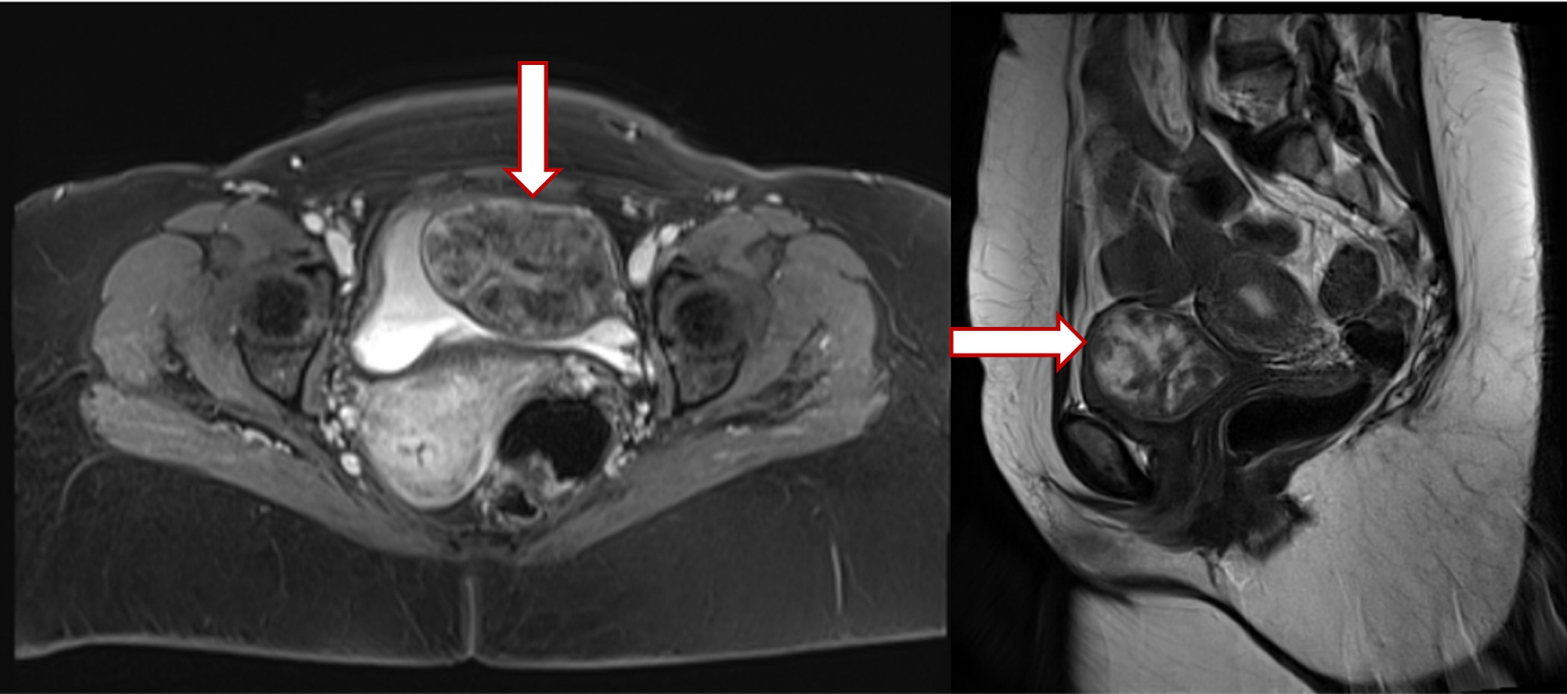
Large submucosal urinary wall bladder tumor, without infiltration into the adjacent organs and tissues.

A 50-year-old, non-smoking female patient with a medical history of diabetes mellitus (DM) and hypertension (HTN) presented to the Urology Department of King Fahad General Hospital with a three-month history of hematuria. She denied any history of flank pain, dysuria, urgency, fever, or weight loss, or appetite. Clinical examination was normal, laboratory findings were within the normal range, urine analysis showed >40 red blood cells (RBCs) per high power field, and urine culture showed no bacterial growth. Bladder smooth muscle cell markers (smooth muscle actin (SMA), desmin, and calponin) were positive. B-catenin and CD117 were negative. An abdominal and pelvic CT scan with contrast was done and showed a large submucosal urinary bladder mass. An abdominal/pelvic MRI was performed and revealed a large submucosal well-defined round mass with a slightly lobulated smooth margin originating from the submucosal layer of the left anterolateral wall of the urinary bladder. It measured 5 x 7 x 4.5 cm and was isointense to the T1 weighted image's muscle and heterogenous with the cystic component on the T2 image. No suspicious lymph nodes in the pelvis and no infiltration into the adjacent organs and tissues were found. The finding suggests bladder leiomyoma and solitary fibrous tumor (Figure 1).

**Figure 2:**
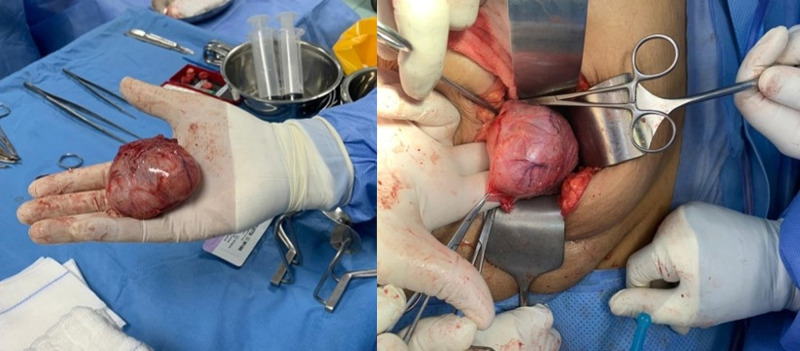
Large submucosal mass.

**Figure 3:**
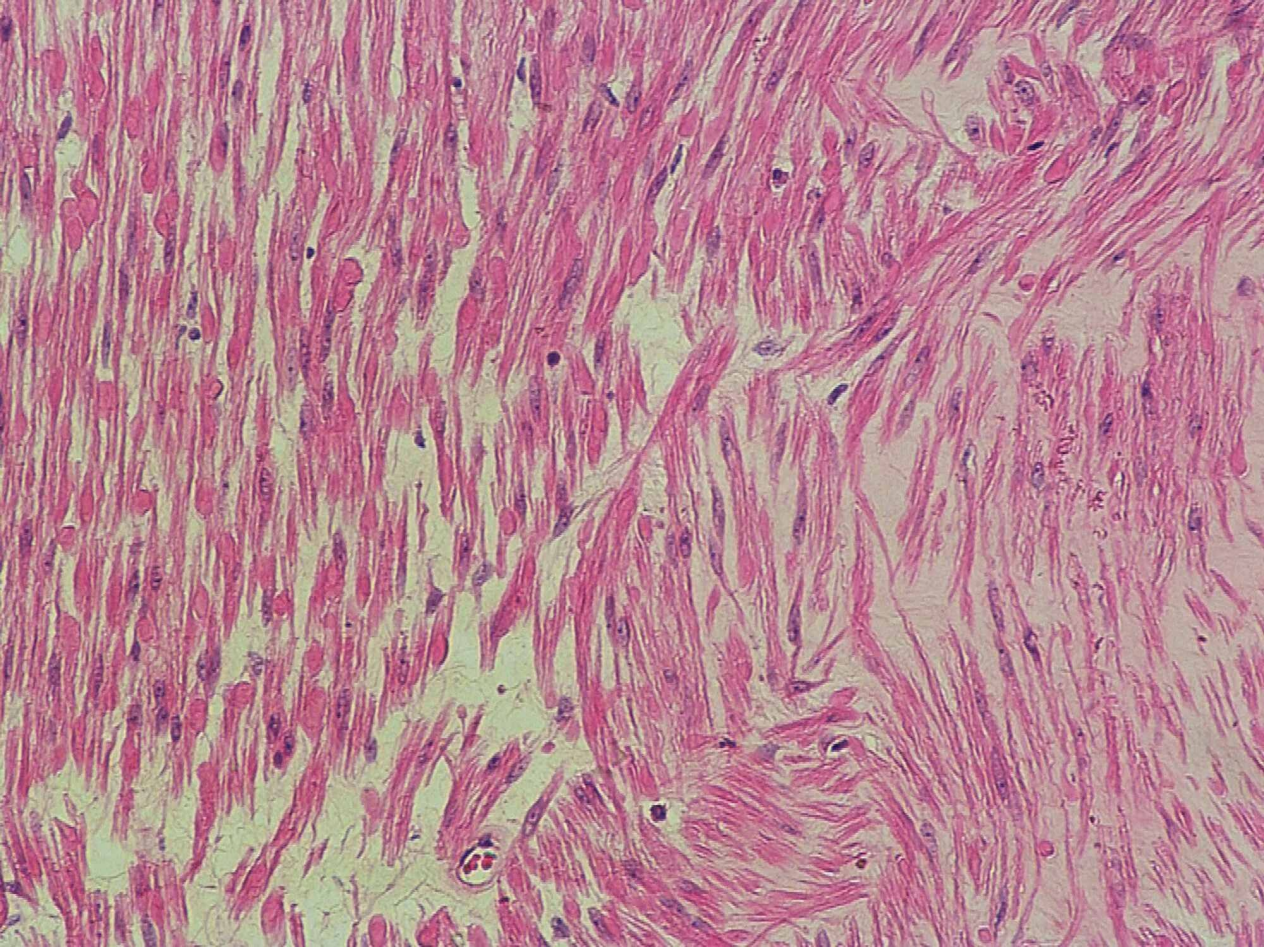
The histopathological examination showed spindle cells in sheets which is pathognomonic for leiomyoma.

An ultrasound-guided biopsy sample was taken and sent for histopathological examination and revealed a spindle cell tumor. On cystoscopy, a normal urinary bladder wall with no obvious exophytic masses and both ureteric orifices were seen, and a urine jet was present, but there was a massive indentation in the left posterolateral site of the urinary bladder extending to the dome of the bladder. The patient then underwent an open partial cystectomy (Figure 2). A specimen of 7 x 5 x 3.5 cm was obtained and preserved in formalin for histopathological examination. A well-encapsulated white firm mass measuring 3 x 2.5 cm was found and the histopathological examination showed spindle cells in sheets which confirm the diagnosis (Figure 3).

**Figure 4:**
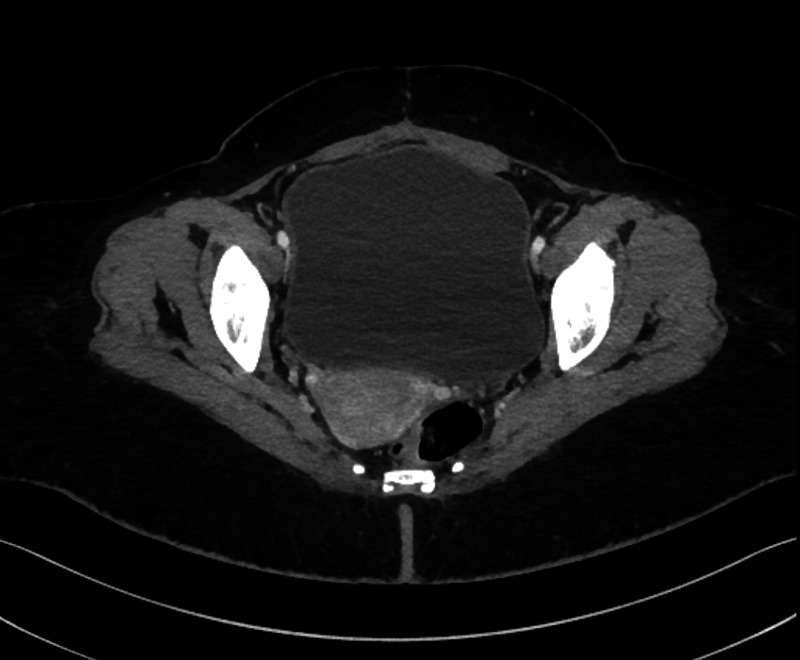
CT abdomen and pelvis with IV contrast at one and half year follow-up.

A cystogram was done in post-operative day 8 and confirmed no urine leak. The Foley catheter was removed, and the patient was discharged in good condition without any complains. In four months follow-up, cystoscopy showed normal both ureteric orifices with normal bladder wall and no obvious bladder masses. CT abdomen and pelvis with IV contrast in delayed images for follow-up after one and half year showed contrast in the urinary bladder with no definite filling defects (Figure 4). In the three, six months and one-year follow-ups, the patient was evaluated, and no hematuria or other complains were recorded.

## Discussion

Benign mesenchymal tumors of the bladder consist of rhabdomyomas, leiomyomas, fibromyomas, fibromas, and osteomas. A leiomyoma is the most common histological type of benign mesenchymal tumor of the bladder among these types, which is commonly found in middle-aged females. In most cases, these lesions could be endovesical (63%) and are rarely extravesical (30%) or intramural (7%) in location [2]. Urinary bladder leiomyoma symptoms depend on the tumor’s size and location. It usually presents with obstructive urinary symptoms (49%), irritative symptoms (38%), flank pain (13%), or hematuria (11%). Tumors arising near the bladder neck or ureteral openings are more likely to cause obstructive symptoms, while larger tumors tend to cause irritative symptoms [4]. Imaging techniques for diagnosing bladder leiomyomas include ultrasound, CT, and MRI. Pelvic ultrasound can detect a homogenous mass. Abdominal CT determines the location of leiomyoma in the bladder lumen, and enhanced CT can further show the tumor's variable degrees. MRI is better than CT for detecting the origin and distinguishing tumor boundaries [1]. However, a histopathological study is a gold standard for diagnosis. Primarily, the tumors' dimensions and anatomical location determine treatment and prognosis. Surgical excision has a good prognosis and should always be offered. Moreover, small and easily accessible tumors can be treated effectively with TURBT [4]. In the case of large tumors and those with extravesical growth or a tumor with an unfavorable position and difficulty in recognition, open surgery with segmental resection or partial cystectomy is usually required, as in our case.

## Conclusions

A bladder leiomyoma is a rare, benign disease with a good prognosis. In our experience, partial cystectomy is the treatment of choice. In case of submucosal tumors, TURBT is the mainstay of treatment for small and easily accessible tumors. However, in cases of unfavorable position and difficulty in recognition, open partial cystectomy might be required as in our case.
